# Clinical Issues for Pediatric Pulmonologists Managing Children With Thoracic Insufficiency Syndrome

**DOI:** 10.3389/fped.2020.00392

**Published:** 2020-07-23

**Authors:** Gregory J. Redding

**Affiliations:** Pulmonary and Sleep Medicine Division, Department of Pediatrics, Seattle Children's Hospital, University of Washington School of Medicine, Seattle, WA, United States

**Keywords:** spine, thorax, pulmonary, scoliosis, management, children

## Abstract

Thoracic insufficiency Syndrome (TIS) is a recently coined phrase to describe children with spine and chest wall deformities, inherited and acquired, who have respiratory impairment, and are skeletally immature. This population has both restrictive and less often obstructive lung disease due to changes in spine and rib configuration which reduce lung volume, stiffen the chest wall, and reduce respiratory muscle strength. Although the population is heterogeneous with regard to age of onset, etiology, severity of deformity, and rate of progression of the deformity, there are common issues that arise which can be addressed by pediatric pulmonologists. These are illustrated in this review by using Early Onset Scoliosis as a common form of TIS. The pulmonary issues pertaining to TIS require collaboration with multi-disciplinary teams, particularly spine surgeons, in order to make decisions about non-surgical and surgical strategies, timing of surgery and medical supportive care over time. Pulmonary input about respiratory function should be used in conjunction with structural features of each deformity in order to determine the impact of the deformity and the response to various treatment options. In those patients with residual lung function impairment as young adults, pediatric pulmonologists must also ensure successful transition to adult care.

## Introduction

Thoracic Insufficiency Syndrome (TIS) is defined as impairment in breathing and/or postnatal lung growth due to spine and thoracic cage deformity in children who are skeletally immature (1). It is defined by abnormal respiratory function but the specific indices used to identify impairment have not been standardized. Skeletal maturity is defined radiographically by the closure of growth plates in certain bones and indicates that further growth and hence spine and thoracic deformities that progress with growth are less likely to worsen. There is an international registry of more than 8,000 children at risk for TIS maintained by the Pediatric Spine Study Group (2). As of 2019, <5% of the patients in this registry had interpretable pulmonary function data. Pediatric pulmonologists are not often intimately involved with the clinical decision making in the management of these patients. Yet their input can be of great value to surgeons, primary care providers, and families regarding the pulmonary status and functional impact of spine and chest wall deformities over time and with treatment.

This chapter addresses the input that pulmonologists can provide at all ages in children with EOS as an example of TIS, as part of an multi-disciplinary team, which ideally includes spine surgeons, pulmonologists, general surgeons, neurosurgeons, pediatric sleep specialists, nutritionists, genetic counselors, physical therapists, and bioethicists. The management of children with TIS begins on first encounter and ends for many with transition to adult care, i.e., over 15–20 years with multiple surgical procedures throughout that time. Pediatric pulmonologists are ideally involved with management questions that arise throughout this time period.

## TIS and Etiologies of EOS

TIS is often recognized in the newborn period by respiratory distress associated with hypoplastic thoraces due to inherited skeletal dysplasias. Small chest walls are classified by surgeons as thoracic “volume depletion” disorders due to the small size of the chest wall. Classic examples are Jeune's syndrome (asphyxiating thoracic dystrophy) and Jarcho-Levin syndrome (which includes both spondylocostal dysostosis and spondylothoracic dysplasia). Khombourlis has reviewed the more than 100 different skeletal dysplasias that can produce respiratory impairment in infancy (3). Many of these are lethal without invasive respiratory support in the first year of life. Several surgical techniques to enlarge the chest wall, such as sternal struts, rib-to-rib attachments, and rib-based expandable titanium arcs have been used in small case series to improve lung volumes. A recent review highlights the surgical options for children with hypoplastic thoraces (4). However case series reporting long-term outcomes are rare and not generalizable to all conditions.

**Figure 1:**
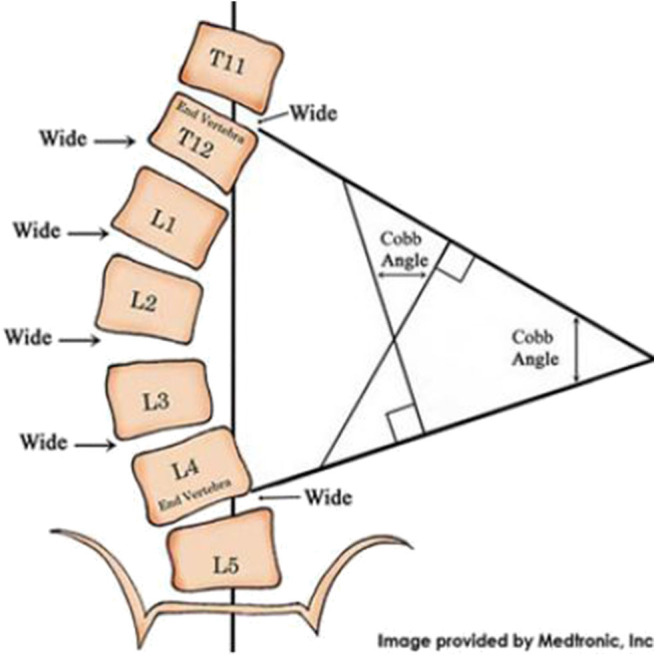
Measurement of the spine's coronal curve using the Cobb angle: Lines drawn along the edges of the vertebrae that are most angled relative to a horizontal line above and below the apex of the curve. The intersection of those lines is used to derive the Cobb angle.

A common condition producing TIS is early onset scoliosis (EOS) which presents before 10 years of age with a coronal curve spine curve >10 degrees. The coronal degree of spine curvature is measured using the Cobb angle, illustrated in Figure 1. The Cobb angle (and its progression over time) is the primary structural measure used by spine surgeons to make decisions about surgical and non-surgical interventions such as casting and bracing.

The etiologies for EOS include congenital, syndromic, thoracogenic, neuromuscular, and idiopathic scoliosis. *Congenita*l scoliosis is defined by the presence of vertebral and rib structural deformities such as vertebral hemi-vertebrae and failure of segmentation with fused or block vertebrae. These may be associated with multiple fused ribs which either constrain chest wall motion or provide large gaps between ribs that may produce a flail chest syndrome. Many *syndromes*, e.g., VACTERL syndrome, have multiple organ involvement and decisions must be made what to treat first. Up to 12% of children with congenital scoliosis also have congenital heart disease, and these children represent an overlap between congenital and syndromic categories (4). Children with *thoracogenic* scoliosis are those receiving thoracic surgery at an early age, either for diaphragmatic hernias, rib resections with tumors, pneumonectomies, or even cardiac repair (5). Up to 30% of children with congenital diaphragmatic hernias will develop subsequent scoliosis (6). This is compounded by pulmonary hypoplasia, which is worse on one side. Up to 10% of children undergoing thoracotomy for congenital heart disease will develop scoliosis.

The most common form of TIS due to EOS is scoliosis associated with *neuromuscular* diseases that produce spasticity or weakness. More than 90% of children with spinal muscular atrophy types I and II will develop scoliosis (7, 8). The frequency of scoliosis among children with cerebral palsy varies among reports from 5 to 80% (7). In one large series of 666 children, 17% had mild scoliosis and another 17% had moderate to severe scoliosis (9). Those with more severe cerebral palsy, based on GMFCS levels of 3–5, had a 50% prevalence with age of onset at 8 years. EOS accounts for <10% of all scoliosis in childhood, with the vast majority presenting after age 10 years with adolescent idiopathic scoliosis.

*Infantile idiopathic* scoliosis begins before age 3 and can vary in severity on presentation. The posterior rib hump or abnormal posture is often the first finding identified by parents. Respiratory concerns are rare initially but may cause failure to thrive due to increased respiratory work with feeding. In young children (<2 years) with infantile idiopathic scoliosis and small coronal spine curves (<30 degrees) there is high rate of reversal to an almost normal spine shape with serial casting treatment over a period of years (10, 11). In these cases, there may be no pulmonary sequelae after orthopedic treatment. However, with larger spine curves, kyphosis, and etiologies for scoliosis other than idiopathic scoliosis, casting/bracing may not be sufficient to correct the deformity, or prevent curve progression and respiratory impairment. Casting is then used as a tactic to delay surgical intervention until the child is older. Thereafter, surgical use of “growth-friendly” expandable distraction rods are used to control scoliosis until pre-adolescence when spine fusion is often undertaken.

The etiology of scoliosis is important as it is one factor that dictates the risk and rate of progression of a spine/thorax deformity over time. Children with fused vertebrae and fused ribs are most likely to progress due to structural deformities (12). Duchenne's muscular dystrophy will produce scoliosis in 90% of boys but does so in young adulthood instead of early adolescence if they are treated early with steroid therapy (13). SMA I and II have new “natural histories” with the advent of nusinersin and adenoviral gene therapy. Scoliosis may present later in these children than previously reported as SMA-specific treatment becomes more common.

The heterogeneity of etiologies producing EOS has made assessments of management strategies, such as surgical and non-surgical interventions difficult to assess and predict. A classification system to address this heterogeneity has been devised for spine surgeons which includes age of onset, etiology of scoliosis, degree of coronal curve magnitude, degree of kyphosis, and rate of progression of the coronal curve deformity over time (14). Use of this classification system has enabled surgeons to estimate risk of post-operative complications (15). However, there are no functional elements in this system, such as pain, nutritional status, or lung function measures. This reflects the frequent lack of effective input by pediatric pulmonologists for these children.

## Pathophysiology and Clinical Consequences of EOS and Other TIS Etiologies

The number of children with EOS that have TIS is uncertain due to the vague nature of the definition of TIS. However, there are common pathophysiologic processes regardless of the etiology. The majority have clinical evidence of restrictive respiratory disease. This is manifested earliest as tachypnea associated with activity or exercise and often is identified by families as exercise intolerance or easy fatigability with exertion. Dyspnea, used in one study to determine the functional consequences of EOS among children old enough to perform spirometry, did not occur frequently until FVC as a % predicted using arm span for height fell below 50% (16). Children with restrictive chest wall disease often adapt by reducing the intensity of their daily activities. They instead become sedentary to avoid dyspnea with activities. Restrictive changes have been measured both as reduced FVC with a normal FEV1 or alternatively by measuring Total Lung Capacity (TLC). Restrictive respiratory mechanics are produced by reduced chest wall compliance due to deformity and perhaps an additional reduction in lung compliance based on reduced lung volume in various lung regions. The reduction in chest wall compliance seems intuitive but data demonstrating this is rare. Motoyama measured total respiratory compliance in children with EOS during anesthesia and found it to be low prior to surgery and lower after titanium struts were attached to the chest in an effort to straighten the spine (17).

Decreases in total respiratory compliance leads to an increase in respiratory work and caloric expenditure and hence a reduced weight for age or body mass index (using arm span for height). Children will often avoid large meals and graze, eating frequent small volume snacks. Failure to thrive is reported in 50% of children with EOS and responds in most cases to caloric supplementation either by mouth or with a feeding tube or gastrostomy tube (18).

**Figure 2:**
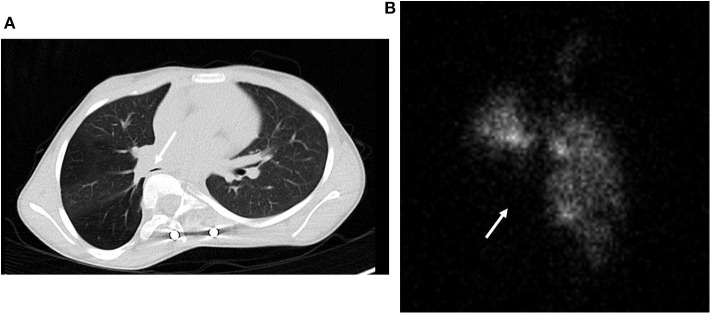
**(A)** CT scan of lobar bronchial compression by the spine due to scoliosis and vertebral intrusion into the thorax. **(B)** Lung ventilation scan correlated with the CT scan in **(A)** demonstrating loss of ventilation in the right middle and lower lobes.

Obstructive lung disease occurs in 10–30% of children with EOS (19, 20). In children with EOS who demonstrated reduced FEV1/FVC or reduced FEV1 as a percent of normal, one third had airway reactivity due to asthma (21). Consequently, if obstructive lung disease is present in TIS, a bronchodilator challenge is in order. In at least 2/3 of cases, the airway obstruction is not reversible with a bronchodilator and usually reflects compression of a lobar or mainstem bronchus by intruding spinal elements and mediastinal structures, as illustrated in Figure 2A. where the right lower lobe bronchus is compressed. Distal fixed tracheal obstruction also occurs by this mechanism. These central airway narrowings are documented by direct visualization during bronchoscopy or alternatively by CT scan images. If a subcarinal airway is compressed, the degree of obstruction described by spirometry will underestimate the severity of the local airway obstruction.

Distortion of the thorax and spine deformity can alter the distribution of ventilation and perfusion quantitated by nuclear lung scans. Unless there is a history of past lung or airway insults, such as pneumonia, the distribution of ventilation and perfusion closely track one another in children with EOS. However, more than half of children with EOS have asymmetric function with one lung functioning better than the other (22). This can be extreme, i.e., 90 vs. 10% in the two hemi-thoraces (22). The function on the concave side of the spine curvature is reduced 60% of the time and relative function of the right and left lung cannot be discerned from a chest radiograph. Figure 2B depicts the ventilation lung scan and loss of ventilation due to right lower lobe compression by the rotated vertebrae portrayed in Figure 2A. Surgical management in this case focused on recovery of lung function in the lower lobe with movement of the vertebra posteriorly with a growth friendly expandable titanium rod. The implications of asymmetric lung function have not been reported. However, surgeons are aware that interventions on the side with the greatest function, particularly if it requires a thoracotomy, predispose the child to respiratory failure post-operatively. In addition, there is speculation that the poorly ventilated lung may be predisposed to atelectasis if lung inflation during sighing is compromised. This could predispose children with severe scoliosis to prolonged atelectasis and need of augmented mucus clearance therapy with infections.

**Figure 3:**
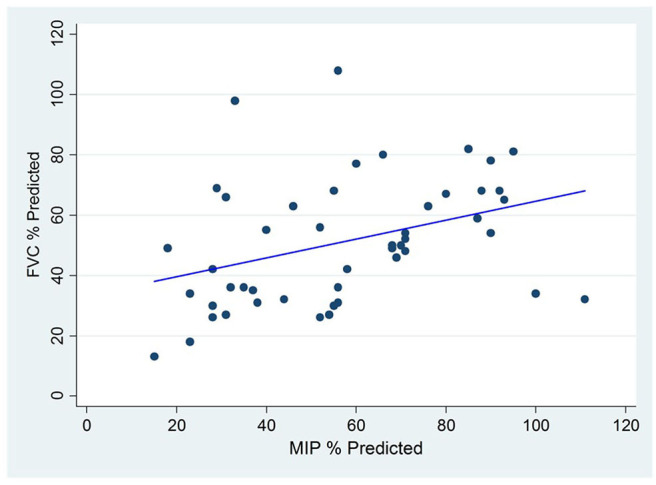
Correlation between Maximum Inspiratory Pressure (MIP) and Forced Vital Capacity (FVC) among Children with Early Onset Scoliosis without underlying neuromuscular conditions.

There is also evidence of reduced respiratory muscle force generation involving both inspiratory and expiratory muscles in children with EOS. As the ribs become crowded due to spine deformities and the costo-vertebral joints become ankylosed with prolonged immobility, the function of some intercostal muscles is reduced and patients become more dependent on diaphragm function to accomplish inspiratory work. There is less sharing of elastic loads among all the respiratory muscles during time of illness and therefore an increased risk of respiratory muscle fatigue and hypercarbic ventilatory failure. A similar event occurs during REM sleep when intercostal tone and function are diminished. Like children with primary neuromuscular weakness, children with EOS experience periods of hypercarbia during sleep before demonstrating daytime hypercarbia. The mechanisms for poor respiratory muscle performance are unclear. Respiratory muscle weakness involves both inspiratory and expiratory muscles as manifested by reduced Maximum Inspiratory pressures (MIP) and Maximum Expiratory pressures (MEP) in children with EOS and correlation with loss of vital capacity (Figure 3) (23). Reduced MIP is thought to occur due to malposition of the diaphragm and perhaps tethering of the diaphragm as the spine and ribs distort and rotate. It is unknown if the diaphragm muscle fibers *per se* are atrophied or adapt to chronic mechanical loads. In animal models of EOS produced early in life, the diaphragms in adulthood have reduced cross-sectional surface area (24).

The reduced MEP is explained in part by the reduced total lung capacity, the lung volume at which MEP is generated. Reduced TLC is the hallmark of restrictive respiratory disease and the further it is reduced, the more MEP is compromised. Of interest, MEPs tend to be reduced to a greater degree than MIPs although both correlate with diminished vital capacity, as illustrated in (Figure 3). Reductions in both MIP and MEP occur in the absence of underlying neuromuscular weakness conditions but are aggravated when underlying neuromuscular weakness disorders are present. The reduced MEP may lead to reduced force generation during cough and impaired mucus clearance when airway secretions increase, as with infection. Therefore, mucus clearance therapies become part of care when children with severe EOS become ill.

The conditions in which respiratory reserve is needed include sleep, exercise, and illness. Sleep related breathing disorders have been reported in over 90% of 63 children studied in one spine center (25). This high prevalence likely reflects underuse of polysomnograms to assess breathing during sleep in children with EOS and a lack of standardized criteria to refer children with EOS to assess breathing during sleep. The most common features on polysomnograms in this group of children were prolongation and recurrence of hypopneic events during REM sleep and not obstructive apnea *per se* (26). These hypopneic events are often associated with hypoxemia and hence counted in the Apnea Hypopnea Index used to score “obstructive sleep apnea.” Loss of upper airway dilator muscle tone during REM sleep may also contribute to hypopneas. The tendency to become hypoxemic with these events may be related to low lung volumes due to small chest wall size and/or regional chest wall deformity with lung distortion. Regional lung constraint at low lung volumes may lead to increased airway closure and thus predispose to hypoxemia during sleep. Up to 25% of children with EOS in one study were found to have higher than normal hemoglobin concentrations (27). It is likely that sleep related recurrent hypoxemia and increased erythropoietin account for these findings.

Sleep related breathing disorders in children with EOS are aggravated by the adenoid and tonsillar enlargement that occur in normal children. An upper airway examination should not be overlooked in these children. Although few patients have been reported, BIPAP ameliorates sleep-disordered breathing more often than oxygen treatment alone in children with EOS (25).

Exercise tolerance is also impaired by TIS. In one report of 35 children with EOS, the distance walked over 6 min was 10% of predicted values (28). During formal cardiopulmonary exercise testing in more functional patients with EOS, the maximum level of work and maximum oxygen consumption were reduced in proportion to FVC. Sense of dyspnea during exercise also correlated with FVC although children often complained of leg fatigue more than shortness of breath (29). Both tests are impacted by more than respiratory function and include the effects of nutritional status, balance, deconditioning, and cardiac function.

Pulmonary hypertension has been described in adolescents dying of severe scoliosis in the last century. Cor pulmonale is rare in children in whom breathing during sleep has been assessed and treated. Presence of pulmonary hypertension is reported as <10% of children with EOS but should be screened in those with severe spine and thoracic deformities.

These physiologic changes do not correlate with structural features of EOS. Cobb angle correlates poorly with measures of vital capacity, apnea-hypopnea index during sleep, and MIP (23, 25, 26). Glotzbecker also evaluated spine height and thoracic width as predicted by pelvic width among 121 children with EOS and found that these structural elements only explained 25% of the variation in forced vital capacity (30). There is a long-standing interest in developing an algorithm from thoracic and spine structure that could be used as a surrogate for lung function. To date, this has not happened and consequently surgeons are encouraged to measure lung function directly. This does not occur without some pulmonary guidance as to the meaning of the results in a clinical context and the quality of the measurements. Many pulmonologists are content to simply interpret the spirometric results without relating them to the clinical status of the patient and this is a disservice to the spine surgeon community.

Half of EOS spine registry patients were initially encountered at <5 years of age (2). All of the aforementioned pathophysiologic features can occur in children who are too young to perform lung function tests. Respiratory rates, lung perfusion scans, polysomnograms, blood gas tensions, and echocardiograms do not require cooperation and are equally useful in children <5 years old. However, these are not surrogates for measures of lung mechanics which are followed with spirometry. Additional clinical features, based on the physical exam may also prove useful. These include failure to thrive when other etiologies are excluded, asymmetric breath sounds on physical exam, and serial respiratory rates over time. Presence of hypotonia or spasticity are useful indicators of neuromuscular scoliosis. Infant lung functions have been performed and reported in a small series of infants using raised volume forced expiration methods under sedation and under anesthesia (2, 31). Reduced lung volumes have been reported in children <2 years of age with EOS (31). However, these methods are not available in many pulmonary and spine centers. Also, passive lung mechanics do not account for reduced respiratory muscle function and may under-represent the degree of lung function impairment when awake. The relative frequency and sequence of changes in various measures of lung function among children <5 years old with TIS and EOS have not been correlated in a systematic way. It is likely that the cumulative number of pulmonary abnormalities that are identified by physical examination an laboratory tests indices that the spine and chest wall deformities are causing more respiratory impairment but this has not been studied. Children with EOS <5 years old remain a challenging group and no serial measures of objective findings have been described to estimate progressive impact of the thoracic deformity on respiratory function.

## Specific Pulmonary Questions Relevant to Children With TIS and EOS

### How Severely Is Breathing Impaired on First Encounter?

Lung function testing is of great use during the initial encounter of the child with a spine and thoracic cage deformity. FVCs on initial encounter among 100 children with EOS from 5 to 15 years of age ranged from 18 to 89% of predicted using arm span or ulnar length for predicted height to normalized spirometric values (32). This range of values was not related to age. The initial lung functions prior to any surgical treatment are extremely significant as they predict eventual values in adulthood and decline in lung function later in adulthood (33). To date, there are no spine surgical strategies that predictably improve lung function for children with EOS (34, 35). This in part reflects the lack of pulmonary data before and after spine growth modulation treatments and before and after non-invasive spine distraction methods using expandable titanium rods. There is data that among children with TIS due to EOS who require invasive or non-invasive mechanical ventilation for all or some part of the day/night. Twenty-four percent of 77 children in one series were able to reduce their respiratory support after spine surgery (36).

### What Other Pulmonary Conditions Are Impacting Pulmonary Function Results in Children With EOS?

Surgeons assume that lung functions performed by children with TIS represent the impact of the spine and chest wall deformity they are asked to treat. However, to do this, the pulmonologist must first identify confounding conditions such as recurrent aspiration syndrome, asthma and lung injury from previous pulmonary infections and minimize their impact on lung function. Aspiration syndromes due to dysphagia associated with fatigue and work of breathing during eating should be considered in infants with TIS and failure to thrive. Among children with underlying neuromuscular weakness, MIP and MEP measures can be reduced by both the neuromuscular weakness and by the subsequent development of a chest wall deformity. In many cases of spinal muscular atrophy I and II, progressive restrictive chest wall disease occurs even after the deformity is controlled by spine fusion due to further collapse of the chest wall. This has been described as a “parasol chest,” based on the shape of an umbrella or parasol as it is closed (37).

In boys with Duchenne muscular dystrophy followed longitudinally, lung function may decline faster among those developing scoliosis, which imposes restrictive forces on a respiratory system already impaired by weak respiratory muscles (38). Whether spine fusion in this group of children slows the decline in respiratory function due to progressive muscle weakness is unclear. There is no evidence that spine fusion will actually improve lung function in these children with TIS but it may prevent a more rapid progression of the restrictive process (39). The challenge for the pulmonologist is to maximally reverse any process that could affect lung function apart from the structural deformity of the spine and chest wall before any surgical spine procedure is performed. This may not be possible. A good example is the child with unilateral lung hypoplasia associated with congenital diaphragmatic hernia. Up to 30% of children after will develop scoliosis years after repair of the diaphragmatic hernia (6). One lung will function worse than the other due to the relative degree of unilateral hypoplasia. Scoliosis produces a spine curve that is usually concave to the hypoplastic side. Whether the spine shape worsens the function in the small lung or stretches the convex contralateral lung is difficult to ascertain. Serial lung scans may be of help in conjunction with spirometry to describe why lung function worsens over time and how each lung responds to surgical treatment of the scoliosis. The goal of management of children with this problem is to preserve function of the most functional lung.

There are two conditions that are particularly challenging. The first is those children with underlying skeletal dysplasias who have extremity and pelvic deformities. This precludes the use of arm span or ulnar length to normalize lung function when children perform spirometry. We have used absolute values for serial measurements and are exploring whether pelvic inlet width might be a useful normalizing method for children of different size and age. Pelvic width correlates well with spinal height in normal children but may not apply to all children with primary skeletal dysplasias (5).

The other “condition” where pulmonary impairment is difficult to assess is in children between 1 and 3 years of age with EOS treated with serial casting as a way to reverse or hold in check infantile idiopathic scoliosis. Children in casts to treat EOS typically have the entire thorax covered in plaster. The cast itself can limit mobility, modify sleep patterns, and limit caloric intake if the abdominal portion of the cast is not cut out and removed. Placement of the cast is performed under anesthesia and may restrict breathing considerably until the cut-out is performed. Physical examination of the heart, chest, and lungs of children in thoracic casts is limited. Body weight is difficult to interpret especially when a new cast is re-applied every 3–4 months. The pulmonary assessment reflects both the skeletal deformity and the impact of the cast. Ideally the child with EOS receiving casting treatment is assessed in between casting procedures.

### What Respiratory Support and Management Will Improve Lung Function in Children With TIS?

Children with TIS can have failure to thrive, poor sleep quality, and poor mucus clearance when ill. Half of the children with TIS will have a Body Mass Index (BMI) that is <10% of predicted norms using arm span to calculate BMI rather than height (18). This requires attention to nutritional status and the safest way to augment caloric intake long-term. Malnutrition can compromise respiratory muscle function, even in normal individuals. It may also increase wound dehiscence and post-operative infections following spine surgery. In children with TIS and failure to thrive, inadequate caloric intake is common and caloric supplements and/or long-term nighttime drip feedings via a gastrostomy tube are needed to produce a normal weight velocity. Halo traction and spine surgery for EOS can also improve weight gain pre-operatively (40).

Supportive care is also important to assure sufficient high-quality sleep. Arousals from sleep are increased in 45% of children with TIS during overnight polysomnography (25). BIPAP improves both AHI values and oxygenation in children with TIS during sleep. Further study is needed to describe the impact of oxygen, CPAP or BIPAP on sleep fragmentation and arousal frequency in children with TIS. In a small case series of children with TIS, 54% qualified for BIPAP based on an AHI of 5 or more events/hour (25). The impact of scoliosis surgery *per se* on sleep quality in children with EOS has not been reported. As the sleep quality in children with TIS is understudied, it is likely that a portion of these children are also undertreated at night both before and after surgical spine treatment.

### Does Lung Function Predict Post-operative Pulmonary Complications or the Duration of Intensive Care After Surgery?

There is published data that pre-operative pulmonary function and etiology for scoliosis can predict the risk of post-operative complications after spine fusion surgery. Among 298 patients who underwent spine fusion for idiopathic and/or congenital scoliosis, a pre-operative value for FVC <40% tripled the risk for post-operative pulmonary complications (41). Yuan et al. found that among children with EOS, the presence of underlying neuromuscular weakness increased the frequency of post-operative complications requiring >3 days of respiratory support from 15% found in other categories of EOS to 50% (42). Other risk factors include duration of anesthesia (>4 h) and volume of blood transfusions during surgery (43). Post-operatively the need for analgesics to control post-operative pain and thoracoplasty are also risks for longer length of stay after spine fusion in adolescents (44). Certain risks are specific to subpopulations with EOS. For example, pulmonary hypertension is a risk for prolonged intubation following spine fusion for scoliosis among children with congenital heart disease (45).

### What Is the Impact of Spine Surgery on Lung Function?

The pulmonologist must monitor the impact of different surgical strategies using different surgical devices to know when surgery improves or worsens lung function. Such monitoring before and after spine fusion surgery in adolescent idiopathic scoliosis demonstrated that the posterior surgical approach had smaller adverse effects of breathing post-operatively than did an anterior approach or combined anterior-posterior approach to spine fusion (46). This has led to primarily posterior surgical fusion for children with EOS who have more compromised lung function pre-operatively. There is very little data on the long-term changes in lung function during spinal surgical and non-surgical treatments of EOS. In one study, serial measurements of vital capacity in the operating room under anesthesia on children with TIS demonstrated that vital capacity measurements fell 28% despite insertion and subsequent expansions of growing rods over a 6 years period (47). The role of weakened respiratory muscles was not assessed due to the passive pulmonary function testing methods.

In some children, lung function will progressively decline despite spine surgery with growing rods, particularly if progressive spine rotation is a prominent feature of the deformity. Demonstration of progressive loss of lung function may require an alternative surgical strategy, e.g., spine fusion, and this will be dictated by serial lung function measurements interpreted by the pulmonologist.

### What Is the Status of Patients With EOS After All Spinal Surgery Has Been Performed?

Another role of the pulmonologist is to assure that the restrictive pulmonary disease that persists after all surgical treatment has been completed is sufficiently supported medically and that a transition plan is in place as the adolescent with TIS enters adulthood. With the advent of new prosthetic devices for the growing spine in use since 2002, a new population of patients with chronic severe restrictive respiratory disease has emerged in need of adult care. The lung function among EOS “graduates” of spine surgery is worse than among young adults who undergo surgery for adolescent idiopathic scoliosis (48).

Children with severe TIS and/or EOS previously did not survive to adulthood. Adult issues such as occupational risks and risks during and after pregnancy have not yet been addressed in this population. Referrals to adult pulmonologists who are aware of these risks are important. Based on one series of patients with EOS that were re-evaluated 25 years after spine fusion during adolescents, progressive decline in lung function during adulthood is more likely in those with a pre-operative FVC <70% of normal (33).

## Final Thoughts

The field of spine surgery for severe spine and thoracic cage deformities which present early in life is rapidly evolving. There are now spine procedures that modulate or direct spine growth, e.g., spine tethering, staples, and the Shilla procedure (49). These differ from growth friendly expandable rods that reduce spine curvature and foster spine growth through distraction. No pulmonary function testing has been completed with these new spine strategies to know their impact on breathing. The magnetically controlled growing rod which is extended non-invasively is now the most popular method for surgically straightening the spine without resorting to spine fusion in an actively growing child (50). Yet, no lung functions before and after insertion and expansion of these rods has been undertaken. All of these devices make intrathoracic volume larger but do so at the cost of chest wall compliance, which declines with stiff metal rods are attached to the chest (17). The reduced chest wall compliance that normally occurs during childhood, the reduced chest wall compliance of the spine deformity, and the additionally reduced chest wall compliance produced by insertion of growing rods into or next to the spine combine to produce progressive restrictive lung disease over time.

In addition, the timing of surgery depends not only on progressive structural deformity of the spine but also its impact on respiratory function over time. Progressive restrictive lung disease may be an indication for intervention even if the structural progression is not dramatic. Surgical decisions among pre-adolescent children with EOS and progressive spine deformities are also controversial. Spine fusion in this age group curtails further spine growth and hence intrathoracic volume during adolescence but also reduces the frequency of complications related to further spine surgeries if growing devices are used. There is no published pulmonary data to answer this question for each of the etiologies of EOS in early childhood.

There is no surgical procedure yet to salvage lung function impairment in children with TIS or EOS. Doing so remains a goal of care and may require attention to diaphragm function and position and also anterior chest wall reconstruction. Insertion of a Nuss bar to increase chest wall depth in a teenager with severe inoperable congenital scoliosis effectively relieved pulmonary artery compression and pulmonary hypertension but did not improve spirometry (51). New strategies are needed if lifelong restrictive lung disease that may worsen in adulthood is to be avoided. New materials for spine devices may provide more flexibility to maintain chest wall motion, but measurements before and after inserting such devices will be needed to demonstrate improvement. In the interim, there is a new population of children who are emerging with substantial respiratory impairment that require respiratory care along with spine care from the initial presentation, often before age 5 years and extending into young adulthood. This population will require pulmonary expertise to optimize management, assess current and improved spine care techniques, and assure transition to adult care in the future.

## Author Contributions

GR was responsible for the entry of this manuscript.

## Conflict of Interest

The author declares that the research was conducted in the absence of any commercial or financial relationships that could be construed as a potential conflict of interest.
